# Identification of potential therapeutic targets for idiopathic pulmonary fibrosis: an integrated multiomics analysis

**DOI:** 10.3389/fimmu.2026.1754277

**Published:** 2026-06-05

**Authors:** Xingxuan Chen, Shibin Chen, Shuai Zhao, Yupeng Li, Jingkun Chang, Si Shi, Dandan Xu, Lijuan Li, Hong Chen

**Affiliations:** 1 Department of Pulmonary and Critical Care Medicine, The Second Affiliated Hospital of Harbin Medical University, Harbin, Heilongjiang, China; 2 Harbin Medical University, Harbin, Heilongjiang, China; 3 Department of Pulmonary and Critical Care Medicine, Changzhi People’s Hospital, Changzhi, Shanxi, China; 4 Department of Geriatric Respiratory Medicine, Heilongjiang Provincial Hospital, Harbin, China; 5 Department of Pulmonary and Critical Care Medicine, National Clinical Research Center of Respiratory Diseases, China-Japan Friendship Hospital, Beijing, China

**Keywords:** cerebrospinal fluid proteins, drug targets, idiopathic pulmonary fibrosis, mendelian randomization, plasma proteins, therapeutic targets

## Abstract

**Introduction:**

Idiopathic pulmonary fibrosis (IPF) is a progressive and fatal lung disease characterized by persistent inflammation, aberrant extracellular matrix remodeling, and impaired tissue repair. Current antifibrotic drugs can slow disease progression but cannot reverse established fibrosis, highlighting the need to identify novel mechanism-based therapeutic targets.

**Methods:**

We integrated batch-corrected transcriptomic data from the Gene Expression Omnibus with UK Biobank genome-wide association study statistics, including 1,369 cases and 435,866 controls, and proteomic GWAS instruments for plasma and cerebrospinal fluid proteins. Causal associations were evaluated using two-sample Mendelian randomization, Steiger filtering, reverse causality testing, and independent dataset validation. Expression-level validation was performed using bleomycin-induced mouse fibrosis and TGF-β1–stimulated fibroblast models.

**Results:**

Twelve proteins were identified as being associated with IPF risk, including eight pro-fibrotic mediators, such as FN1, CCL5, PPID, and CDON, and four protective factors, including SCARF2, IL7R, ESAM, and CD274. Multi-omics integration and experimental validation prioritized four candidate proteins: SCARF2 as a protective factor, and FN1, PPID, and CDON as pro-fibrotic factors. Network analysis linked FN1 to extracellular matrix remodeling and SCARF2 to scavenger receptor–mediated immune regulation, indicating distinct fibrotic and immunomodulatory pathways.

**Discussion:**

These findings identify several potential therapeutic targets for IPF and provide a translational framework for developing disease-modifying therapies that may overcome the limitations of current antifibrotic treatments.

## Introduction

Idiopathic pulmonary fibrosis (IPF) is a chronic, progressive fibrotic disease of unknown etiology characterized by the gradual accumulation of extracellular matrix proteins, ultimately leading to respiratory failure and death (1). Epidemiological data indicate that the global incidence of IPF ranges from 0.09 to 1.30 per 10, 000 individuals and has been increasing annually. The median survival following diagnosis is only 2 to 3 years (2). Currently, in addition to lung transplantation, antifibrotic agents such as pirfenidone (PFD) and nintedanib are recommended in clinical guidelines for IPF treatment (3, 4). Although both drugs can alleviate symptoms, improve quality of life, and slow the decline in lung function, they do not cure the disease (5). Furthermore, issues related to drug tolerance limit dose adjustments and alternative therapies, highlighting the critical need to identify novel therapeutic targets capable of preventing IPF onset or delaying its progression.

Incorporating genetics into drug development may be one of the most effective strategies to enhance this process, as genetically supported therapies are more likely to succeed in clinical trials (6, 7). Proteins encoded by druggable genes have become targets for existing drugs or potential targets for small molecules or monoclonal antibodies (8, 9).Mendelian randomization (MR) is a powerful tool in epidemiological research that employs genetic variants as instrumental variables to infer causal associations between risk factors and specific diseases (10). In MR studies, genetic variants adhere to the principle of random allelic assignment to offspring, akin to the design of randomized controlled trials, thereby minimizing confounding and reverse causality inherent in observational data (11). This method has become particularly valuable for assessing the causal effects of drug targets on disease outcomes (12, 13). Human proteins, central to diverse biological functions, constitute the predominant category of therapeutic targets (14). Nelson et al. (15) reported that proteins with genetically supported roles as drug targets are twice as likely to secure regulatory approval for clinical use. Concurrently, advancements in high-throughput sequencing technologies have facilitated the generation of large-scale genomic datasets. Integration of such datasets with bioinformatics methodologies enables comprehensive dissection of the genetic and molecular mechanisms underlying disease pathogenesis (16). Thus, synergizing human genetic data, genomic sequencing, and systems biology approaches is critical for elucidating the genetic architectures driving human pathophysiology. However, research applying MR frameworks that combine protein quantitative trait locus (pQTL) data with bioinformatics strategies to explore drug targets for idiopathic pulmonary fibrosis (IPF) remains sparse to date.

**Figure 1:**
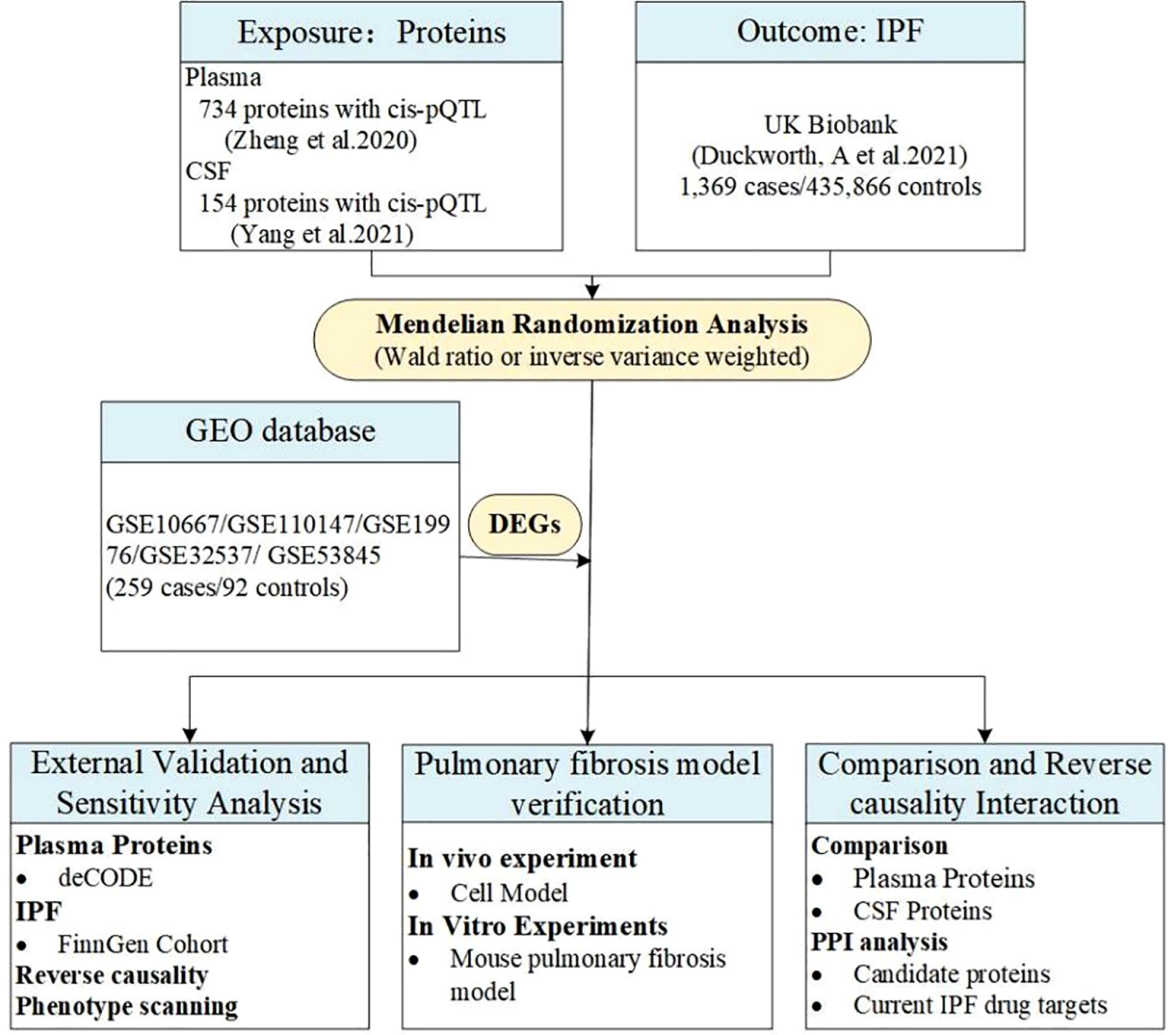
Study design for the identification of plasma and brain proteins causally associated with IPF.

In this study, we integrated plasma and cerebrospinal fluid (CSF) proteomic data with genomic information using Mendelian randomization and comprehensive bioinformatics approaches to identify novel therapeutic targets for idiopathic pulmonary fibrosis (IPF). Specifically, we first utilized genome-wide association study (GWAS) data from the UK Biobank (17), plasma pQTL data reported by Zheng et al. (18), CSF pQTL data from Robins et al. (19), and five IPF-related gene expression datasets from the National Center for Biotechnology Information (NCBI) Gene Expression Omnibus (GEO) to screen for candidate drug targets. Second, key findings were further evaluated through reverse causality tests and phenome-wide association analyses. Third, we validated the expression patterns of the identified genes in pulmonary fibrosis using both *in vivo* and *in vitro* experimental models. Fourth, protein–protein interaction (PPI) networks were constructed to characterize the functional features and biological relevance of these candidate targets. Finally, we conducted external validation using recently published plasma pQTL datasets, including the Finnish database (20) and the study by Ferkingstad et al. (21), to strengthen the robustness of our conclusions. The overall study design is summarized in **Figure 1**.

In summary, our study provides important insights into the discovery of novel therapeutic targets for idiopathic pulmonary fibrosis (IPF). By integrating Mendelian randomization, bioinformatics analyses, drug prediction, phenome-wide scanning, protein–protein interaction network construction, external validation, as well as *in vivo* and *in vitro* experiments, we offer valuable guidance for the development of more effective and targeted therapeutic strategies.

## Methods

### Data collection

In this study, we queried the GEO database (https://www.ncbi.nlm.nih.gov/geo/) using the keyword “Idiopathic pulmonary fibrosis” to identify eligible datasets. Our search criteria included: “Entry type” set to “Series, “ “Study type” designated as “Expression profiling by array, “ species limited to humans, and datasets containing a control group. Consequently, five datasets [GSE10667, GSE110147, GSE19976, GSE32537 and GSE53845] were selected for analysis. The raw or processed expression matrices of the five eligible GEO datasets were downloaded and organized using Perl scripts. Platform annotation files were used to convert probe IDs into official gene symbols. When multiple probes mapped to the same gene, the average expression value was used to represent that gene. Genes without valid gene-symbol annotation were excluded before dataset merging. **Table 1** provides detailed information about the datasets included in this study.

**Table 1:** Characteristics of the five GEO datasets.

GEO datasets	Platform	Sample size		Publication years	Regions
		IPF	Normal		
GSE10667	GPL4133	23	15	2009	Pittsburgh
GSE110147	GPL6244	22	11	2018	London
GSE19976	GPL6244	7	8	2010	Oxford
GSE32537	GPL6244	167	50	2011	Aurora
GSE53845	GPL6480	40	8	2014	South San Francisco

### Differential expression gene analysis

R software (version 4.3.2) was employed to read and preprocess datasets GSE10667, GSE110147, GSE19976, GSE32537, and GSE53845 for individual dataset correction. The datasets were then combined, and batch effect correction was performed, followed by differential expression analysis between 92 normal samples and 259 idiopathic pulmonary fibrosis (IPF) samples. Differential genes were identified using the “Limma” package with criteria of |log2FC|≥0 and adjusted P < 0.05. Volcano plots were generated using ggplot2 to visually depict up- and down-regulated genes, while heat maps illustrated gene expression patterns, providing valuable insights to support subsequent analyses.

### Exposure data

Cerebrospinal fluid pQTL (CSF pQTL) data were obtained from a study that reported 274 pQTL for 184 CSF proteins (19). Plasma pQTL data were retrieved from another study (18) that collated 3, 606 pQTL22–26 for 2, 656 proteins from five previously published GWAS data (22–26). The genetic tool variables used for MR analysis were influenced by three MR assumptions (27), so we included only pQTL that met the following criteria: (1) demonstrated a genome-wide significant association with proteins (P < 5 × 10^−8^); (2) both SNPs and proteins were located outside the Major Histocompatibility Complex (MHC) region (chromosome 6: 25.5–34.0 Mb); (3) were independently associated, with linkage disequilibrium (LD) clustering r² < 0.001; (4) explained the maximum total variance at the protein level using genetic instruments; and (5) SNPs were situated within 1 Mb of the transcription start site of the respective protein-coding gene. The proportion of variance explained (PVE) by these genetic instruments for the proteins ranged from 0.12% to 81.9%. Ultimately, 154 cis-pQTL for 154 proteins were included in CSF proteins, and 738 cis-acting SNPs for 734 proteins were included in plasma proteins (Additional file 1: **Supplementary Table 1**). We check the original data to ensure reliability. In addition, corresponding plasma pQTL data (4, 907 plasma proteins measured in 35, 559 Icelanders) were extracted from the study of Ferkingstad et al. (21) for external validation.

### Outcome data

Idiopathic pulmonary fibrosis data were derived from GWAS data from the UK Biobank (17), and included 1, 369 patients of European ancestry and 435, 866 control subjects of European ancestry. Aggregate data for the GWAS analysis are from the IEU Open GWAS project and can be downloaded at https://gwas.mrcieu.ac.uk/. In addition, IPF data used for external validation came from the FinnGen cohort (2, 189 patients of European ancestry and 407, 609 control subjects of European ancestry, https://www.finngen.fi/en).

### Statistical analysis

MR analysis was completed using R package TwoSampleMR version 4.3.1 (https://github.com/MRCIEU/TwoSampleMR). Exposure and result data are imported and reconciled using the R package built-in function (harmonise_data). The strength of genetic variants was assessed using PVE and F statistics (PVE = 2 × Effect Allele Frequency (EAF) × (1−EAF) × beta²; F = PVE² × (N−2)/(1−PVE²)). Cis-pQTLs with F-statistics greater than 10 were considered strong genetic variants. Subsequently, high-variability cis-pQTLs were extracted from GWAS of idiopathic pulmonary fibrosis (IPF), and harmonized with protein genetic instruments. Following this, Steiger filtering and Mendelian Randomization (MR) analyses were conducted. When the instrumental variables in MR comprised only one SNP, the Wald ratio method was employed to estimate the log change in IPF risk per one standard deviation (SD) increase in plasma protein levels. For instrumental variables with multiple SNPs, the inverse variance weighted (IVW) method was used to determine the causal estimate of plasma proteins on IPF (28). Further sensitivity analyses included the Cochran Q-test and MR-Egger intercept test to assess heterogeneity and horizontal pleiotropy of the MR results. The Steiger test was used to investigate whether there was a reverse causal relationship between exposure and outcome. To account for multiple tests, FDR (False Discovery Rate) (P < 0.05) correction was applied to identify significant MR results (29). Next, the genes obtained by MR analysis were cross-verified with the co-expressed genes between DEGs in the GEO database, including up-regulated or down-regulated genes, to further refine the genes to strengthen the reliability of their causal relationship with the disease. For external validation, MR analysis was performed only on identified genes.To prioritize candidate proteins, we used a sequential evidence-integration framework rather than a subjective weighted scoring system.FDR-significant MR proteins were intersected with DEGs from batch-corrected GEO datasets and further evaluated by sensitivity analyses, phenome-wide scanning, external validation, and expression-level validation in fibrotic models. Consistently supported proteins were classified as primary candidates, whereas those with partial support were defined as secondary candidates.

### Reverse causality detection

The IPF data in the main analysis was taken as the exposure data (Additional file 1: **Supplementary Table 2**), and the preliminarily identified genes were taken as the outcome data, and the bidirectional MR analysis was conducted to detect the potential reverse causality. The inverse variance weighted method (IVW), weighted mode (WM), weighted median method (WME), simple mode (SM) and MR-Egger regression method were used to estimate the effect value (30, 31). Generally speaking, in the absence of heterogeneity and horizontal pleiotropy, the results of IVW, that is, the gold standard of MR analysis, will be preferred. P < 0.05 is considered statistically significant. Steiger test results are used as further supplementary verification.

### Phenome-wide scan

In order to further evaluate the horizontal pleiotropy of potential drug targets, we used the screened pQTL as the key word and searched for the correlation with other characters by searching the literature. At the same time, we used “Phenoscanner” and “LDlink” to carry out phenotypic scanning (32, 33) to explore whether SNPs and other traits have genome-wide significance and whether they are related to any known IPF risk factors, including metabolic traits, protein or clinical traits.

### Plasma and cerebrospinal fluid protein comparative analysis

To investigate the correlation between common brain and plasma pQTLs, Mendelian Randomization (MR) analysis was employed to estimate effect sizes, followed by Spearman correlation analysis for further assessment. Additionally, the study examined the influence of various P-value thresholds on the correlation results to determine whether the significance level affected the findings.

### Constructing protein-protein interaction networks

Protein-protein interaction (PPI) networks are composed of proteins linked by interactions, which participate in various processes of life activities such as biological signal transmission, gene expression regulation, energy and substance metabolism, and cell cycle regulation. By assessing and analyzing protein-protein interaction (PPI) networks, researchers can gain a deeper understanding of how certain proteins interact with other proteins within cells. In this study, interactions between significant proteins were investigated using the Retrieval Interacting Gene Search Tool (STRING) database (https://string-db.org/) with a confidence score of 0.4 (34), further revealing PPI results by Cytoscape (V3.9.1) (35). In addition, we summarized drug targets for IPF currently available on the market and explored drugs corresponding to these IPF-associated genes for predicting potential therapeutic drugs based on the corresponding drug targets in the drug library database (https://www.drugbank.ca) (36). The PPI analysis was exploratory and was not intended to infer causality, pathway activity, or cell-cell communication.

### Animal model

Twelve Eight-week-old male C57BL/6J mice were obtained from Charles River Laboratories (Beijing, China). The animals were randomly allocated to either the control group or bleomycin-induced pulmonary fibrosis model group(n=6). The IPF model was induced by intratracheal instillation of bleomycin (MCE, China; Cat. No. HY-17565A) at a dose of 2.5 mg/kg, dissolved in sterile saline, under pathogen-free conditions. Control mice received an equivalent volume of physiological saline. After 21 days, all mice were deeply anesthetized and euthanized by cervical dislocation, after which lung tissues were harvested for subsequent analyses. All experimental procedures were approved by the Animal Ethics Committee of Harbin Medical University (Approval No. YJSKY2024-023) and were performed in accordance with the Declaration of Helsinki.

### Cell culture and treatment

Human lung fibroblasts (MRC-5) were obtained from Pricella Biotechnology (Wuhan, China). Cells were cultured in standard medium supplemented with 10% fetal bovine serum, 100 U/mL penicillin, and 100 mg/L streptomycin, and maintained at 37 °C in a humidified incubator with 5% CO_2_. All cell lines were tested and confirmed to be free of mycoplasma contamination. When the confluence of MRC-5 cells reached 70–80%, the cells were treated with TGF-β1 (10 ng/mL; PeproTech, Wuhan, China) for 48 h, after which they were harvested for further analysis.

### Western blot analysis

Western blotting was conducted to evaluate the expression levels of FN1, collagen type I, and α-SMA in TGF-β1–stimulated fibroblasts. Briefly, MRC-5 cells were lysed on ice using RIPA buffer (Beyotime, Shanghai, China), and protein concentrations were determined with the BCA Protein Assay Kit. Equal amounts of protein (20 μg) were separated by SDS–PAGE and transferred onto PVDF membranes. Membranes were blocked with 5% non-fat milk and incubated overnight at 4 °C with primary antibodies against FN1 (1:500; Proteintech), α-SMA (1:500; Proteintech), and β-actin (1:500; Proteintech). After washing, membranes were incubated with appropriate HRP-conjugated secondary antibodies. Protein bands were visualized and quantified using the Odyssey infrared imaging system.

### Quantitative real-time PCR

qRT-PCR was performed to validate the expression levels of key genes in TGF-β1–stimulated fibroblasts and bleomycin-treated lung tissues. Total RNA was extracted using TRIzol reagent (Invitrogen, Carlsbad, CA, USA). RNA concentration and purity were measured with a NanoDrop 8000 spectrophotometer (Thermo Fisher Scientific, USA). Complementary DNA (cDNA) was synthesized from RNA using a reverse transcription kit (Vazyme, Nanjing, China). Quantitative PCR was conducted on an ABI 7500 FAST system under the following conditions: initial denaturation at 95 °C for 5 min, followed by 40 cycles of 95 °C for 10 s, 55 °C for 15 s, and 72 °C for 20 s. Relative gene expression levels were calculated using the 2^−ΔΔCt method. Primer sequences are provided in the Supplementary Information. (Additional file 1: **Supplementary Table 8**).

### Histology and immunohistochemistry

Mouse lung tissues were fixed in 4% paraformaldehyde for 24 h, dehydrated over 20 h, and embedded in paraffin. Sections were deparaffinized in xylene, rehydrated through graded ethanol solutions, and stained with hematoxylin and eosin (H&E) or Masson’s trichrome (Solarbio, Beijing, China). For immunohistochemistry, sections were pretreated with 3% hydrogen peroxide to block endogenous peroxidase activity and incubated with goat serum to prevent nonspecific binding. The sections were then incubated overnight at 4 °C with primary antibodies. On the following day, HRP-conjugated secondary antibodies (anti-rabbit IgG) were applied, and chromogenic detection was performed using diaminobenzidine (DAB) substrate.

## Results

### Principal component analysis and identification of differentially expressed genes

Five IPF microarray datasets were obtained from the GEO database as experimental groups in this study. These five datasets included a total of 259 IPF patients and 92 healthy controls. **Table 1** provides details about the included datasets.

**Figure 2:**
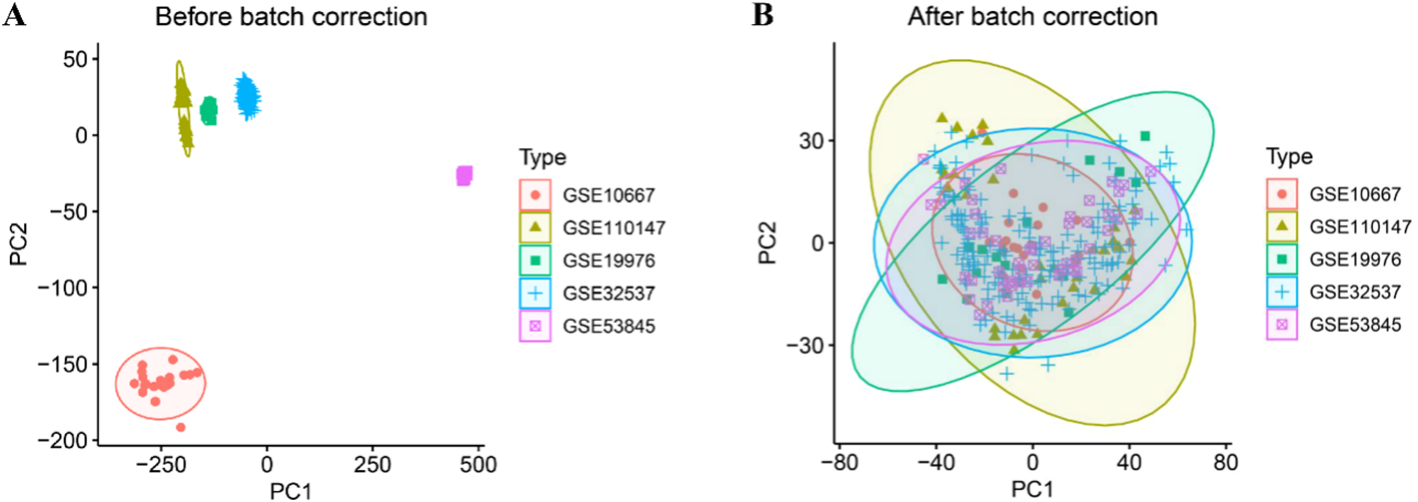
Principal component analysis (PCA). **(A)** Before batch correction. **(B)** After batch correction.

**Figure 3:**
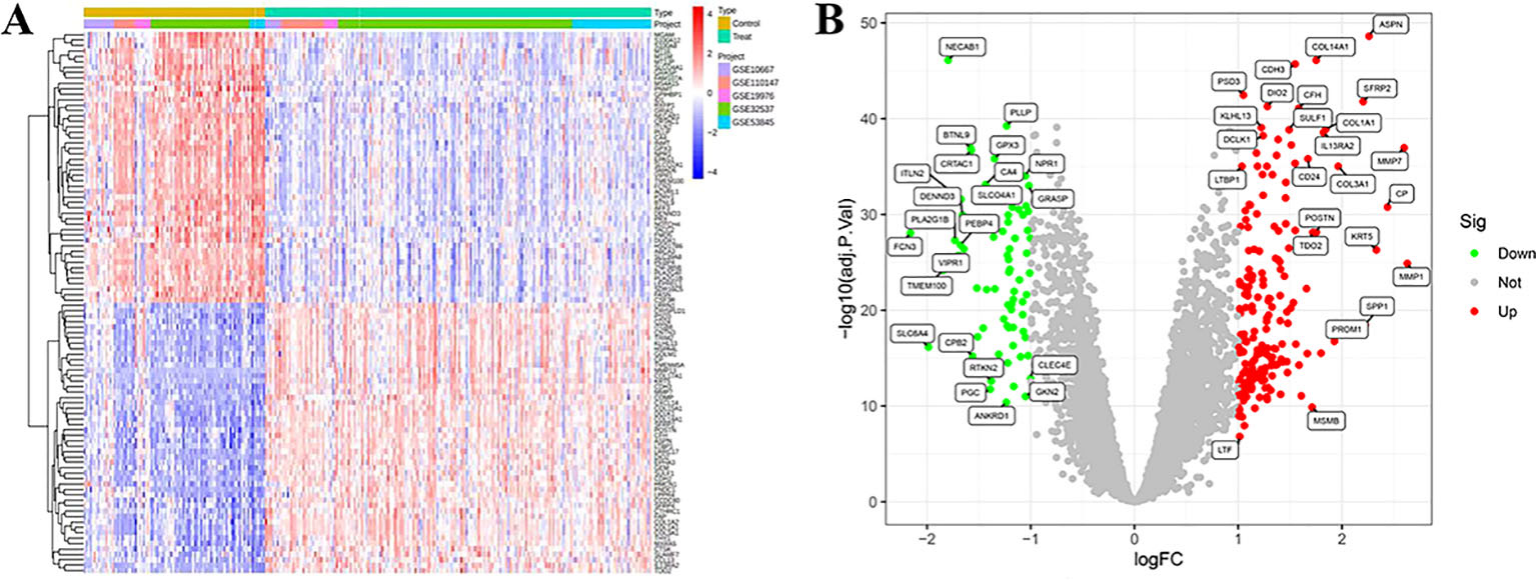
Identification of DEGs in IPF. **(A)** Volcano plot of all DEGs. **(B)** Heatmap of all DEGs.

Using R version 4.3.2, we corrected and combined the expression values for each gene within their respective datasets and mitigated batch effects through principal component analysis (PCA). As illustrated in **Figure 2A**, batch effects were evident across the five IPF gene datasets. Following calibration, as shown in **Figure 2B**, all samples demonstrated acceptable uniformity post-PCA. We identified a total of 256 differentially expressed genes (DEGs), comprising 114 upregulated and 142 downregulated genes (**Figure 3A**). The expression patterns of these DEGs are depicted in heatmaps (**Figure 3B**).

### Screening for IPF-causing proteins in the proteome

MR analysis of 154 proteins in CSF and 734 proteins in plasma revealed that 37 plasma proteins and 12 CSF proteins were significantly associated with IPF susceptibility after FDR correction (P< 0.05) (Additional file 1: **Supplementary Table 3**). Next, we cross-validated the MR analysis results with DEGs. We obtained co-expressed genes between IPF related genes and DEGs (**Table 2**; **Supplementary Figure S1**), including 10 plasma proteins, namely scavenger receptor class F member 2 (SCARF2), fibronectin 1 (FN1), C-C motif chemokine ligand 5 (CCL5), peptidylprolyl isomerase D (PPID), interleukin 7 receptor (IL7R), killer cell lectin like receptor B1 (KLRB1), cell adhesion associated, oncogene regulated (CDON), immunoglobulin superfamily DCC subclass member 4 (IGDCC4), endothelial cell adhesion molecule (ESAM), CD274 molecule (CD274) and two cerebrospinal fluid proteins. Specifically, FN1 (OR = 1.32, 95% CI = 1.07-1.55, P = 2.38×10^-2^), CCL5 (OR = 1.04, 95% CI = 1.01-1.13, P = 1.06×10^-2^), PPID (OR = 1.09, 95% CI = 1.01-1.15, P = 1.60×10^-2^), KLRB1 (OR = 1.02, 95% CI = 1.01-1.04, P = 2.20×10^-2^), CDON (OR = 1.03, 95% CI = 1.01-1.05, P = 4.25×10^-2^), IGDCC4 (OR = 1.21, 95% CI = 1.07-1.31, P = 2.36×10^-2^), and CSF protein TNFSF15(OR = 1.15, 95% CI = 1.02-1.21, P = 2.36×10^-2^), ECM1 (OR = 1.08, 95% CI = 1.01-1.13, P = 1.90×10^-3^) increases the risk of IPF, whereas SCARF2(OR = 0.89, 95% CI = 0.68-0.97, P = 4.13×10^-3^), IL7R(OR = 0.76, 95% CI = 0.48-0.92, P = 5.09×10^-3^), ESAM (OR = 0.77, 95% CI = 0.56-0.87, P = 3.86×10^-2^), an increase in CD274 (OR = 0.85, 95% CI = 0.71-0.91, P = 1.85×10^-2^) was associated with a reduced risk of IPF (**Table 2**). We also performed heterogeneity tests showing no significant heterogeneity or outliers (Additional file 1: **Supplementary Table 4**).

**Table 2:** The cross-validation results of differentially expressed genes (DEGs) in MR analysis showed significant correlation with IPF.

Tissue	Protein	UniProt ID	SNP^a^	Effect allele	OR (95% CI)^b^	P value	PVE	F statistics	Author
Plasma	SCARF2	Q96GP6	rs738086	T	0.89 (0.68, 0.99)	4.13×10^-3^	2.62%	88.82	Sun
Plasma	FN1	P02751	rs1250258	C	1.32 (1.07, 1.55)	2.38×10^-2^	19.78%	245.58	Suhre
Plasma	CCL5	P13501	rs4239252	A	1.04 (1.01, 1.13)	1.06×10^-2^	4.16%	43.25	Suhre
Plasma	PPID	Q08752	rs8396	C	1.09 (1.01, 1.15)	1.60×10^-2^	10.73%	118.8	Suhre
Plasma	IL7R	P16871	rs11957503	G	0.76 (0.48, 0.92)	5.09×10^-3^	8.69%	94.74	Suhre
Plasma	KLRB1	Q12918	rs3933456	C	1.01 (0.99, 1.02)	2.20×10^-2^	2.05%	67.11	Emilsson
Plasma	CDON	Q4KMG0	rs3740909	T	1.03 (1.01, 1.05)	4.25×10^-2^	10.22%	113.09	Suhre
Plasma	IGDCC4	Q8TDY8	rs8034057	G	1.21 (1.07, 1.34)	2.36×10^-2^	2.05%	67.08	Emilsson
Plasma	ESAM	Q96AP7	rs11219769	T	0.77 (0.56, 0.87)	3.86×10^-2^	2.11%	71.01	Sun
Plasma	CD274	Q9NZQ7	rs1411262	C	0.88 (0.71, 0.91)	1.85×10^-2^	3.96%	131.82	Emilsson
CSF	TNFSF15	O95150	rs6478109	G	1.15 (0.99, 1.21)	3.19×10^-2^	12.99%	124.61	Yang
CSF	ECM1	Q16610	rs7002	G	1.08 (1.01, 1.13)	1.90×10^-3^	4.99%	43.86	Yang

PVE, proportion of variance explained.

^a^ All SNPs used were cis-acting.

^b^ Odds ratios for increased risk of asthma were expressed as per SD increase in plasma protein levels and per 10-fold increase in CSF protein levels.

### Sensitivity analysis

Given that Mendelian randomization (MR) analyses can be influenced by pleiotropy of instrumental variables (IVs), we conducted several sensitivity analyses. First, reverse MR analyses were performed for the 10 identified plasma proteins and IPF, which did not reveal any causal effect of IPF on the levels of these plasma proteins. We further confirmed the directionality of the associations using Steiger filtering. Due to the absence of consistent instrumental variables in cerebrospinal fluid (CSF) pQTL-GWAS, reverse MR analyses were not conducted for CSF proteins (**Table 3**; **Supplementary Figure S2**). Second, potential confounding factors for the identified proteins were investigated through literature review, PhenoScanner, and LDlink. Results showed that SCARF2 is associated with lipid regulation, chronic obstructive pulmonary disease (COPD), and idiopathic pulmonary fibrosis (IPF). FN1 is linked to waist-hip ratio, lipid levels, hypertension, and is a known indicator of pulmonary fibrosis. CCL5 has associations with immune cell activity and rheumatic immune diseases, such as phagocytes and leukocytes. Epidemiological studies suggest higher comorbidity rates of IPF with autoimmune diseases, indicating shared genetic factors. PPID is involved in metabolic processes and amino acid transport. IL7R relates to leukocytes, including neutrophils, eosinophils, basophils, and lymphocytes. KLRB1 is associated with primary Sjögren’s syndrome, chronic inflammatory reactions, and tumor invasion; it is also possible that Sjögren’s syndrome may influence the causal relationship between KLRB1 and IPF. IGDCC4 correlates with blood protein levels and immunoglobulin superfamily DCC subclass member 4. ESAM is linked to schizophrenia and vascular permeability. CD274 is associated with blood protein levels and programmed death-ligand 1. TNFSF15 relates to inflammatory bowel disease, Crohn’s disease, and primary biliary cholangitis. ECM1 is associated with atopic dermatitis. No significant associations were observed for CDON (**Table 3**; Additional file 1: **Supplementary Table 5**).

**Table 3:** Summary of reverse causality detection, Steiger filtering and phenotype scanning on twelve potential causal proteins.

Tissue	Protein	UniProt ID	SNP	Bidirectional MR (MR-IVW)^a^	Steiger filtering	Previously reported associations
Plasma	SCARF2	Q96GP6	rs738086	0.920 (0.801-1.057)	Ture 4.80×10-21	Lipid metabolism ^b^ Chronic obstructive pulmonary disease ^c^
Plasma	FN1	P02751	rs1250258	0.987(0.958-1.017)	Ture 1.28×10-49	Hypertension ^b^ Height ^b^ Total cholesterol levels ^b^
Plasma	CCL5	P13501	rs4239252	0.991(0.946-1.037)	Ture 3.78×10-27	C-C motif chemokine 5 levels ^b^
Plasma	PPID	Q08752	rs8396	0.994 (0.947-1.043)	Ture 7.83×10-11	Metabolite levels ^b^ Metabolic traits ^b^
Plasma	IL7R	P16871	rs11957503	0.973(0.941-1.007)	Ture 1.92×10-21	White blood cell ^b^
Plasma	KLRB1	Q12918	rs3933456	0.995 (0.962-1.029)	Ture 6.90×10-28	chronic inflammation ^b^ Sjogren’s syndrome ^c^ Tumor infiltration ^c^
Plasma	CDON	Q4KMG0	rs3740909	1.010(0.974-1.048)	Ture 6.20×10-26	NA
Plasma	IGDCC4	Q8TDY8	rs8034057	0.987(0.922-1.033)	Ture 4.80×10-12	Blood protein levels ^c^
Plasma	ESAM	Q96AP7	rs11219769	0.998(0.962-1.035)	Ture 6.60×10-18	Vascular permeability ^b^ schizophrenia ^c^
Plasma	CD274	Q9NZQ7	rs1411262	1.009(0.974-1.045)	Ture 1.00×10-200	Blood protein levels ^b^ PD-L1 levels ^b^
CSF	TNFSF15	O95150	rs6478109	NA	Ture 3.78×10-27	Inflammatory bowel disease ^c^ Crohn’s disease ^b^ Primary biliary cholangitis ^c^
CSF	ECM1	Q16610	rs7002	NA	Ture 1.36×10-10	Monocyte ^c^ Blood platelet ^b^

MR-IVW, Mendelian randomization with inverse variance weighted method; PP, posterior probability.

^a^ Odds ratios per SD increase in plasma protein levels and per 10-fold increase in CSF protein levels as asthma risk increased.

^b^ SNP associated with traits directly.

^c^ SNP associated with traits mediated by its proxy.

### External validation of IPF

**Figure 4:**
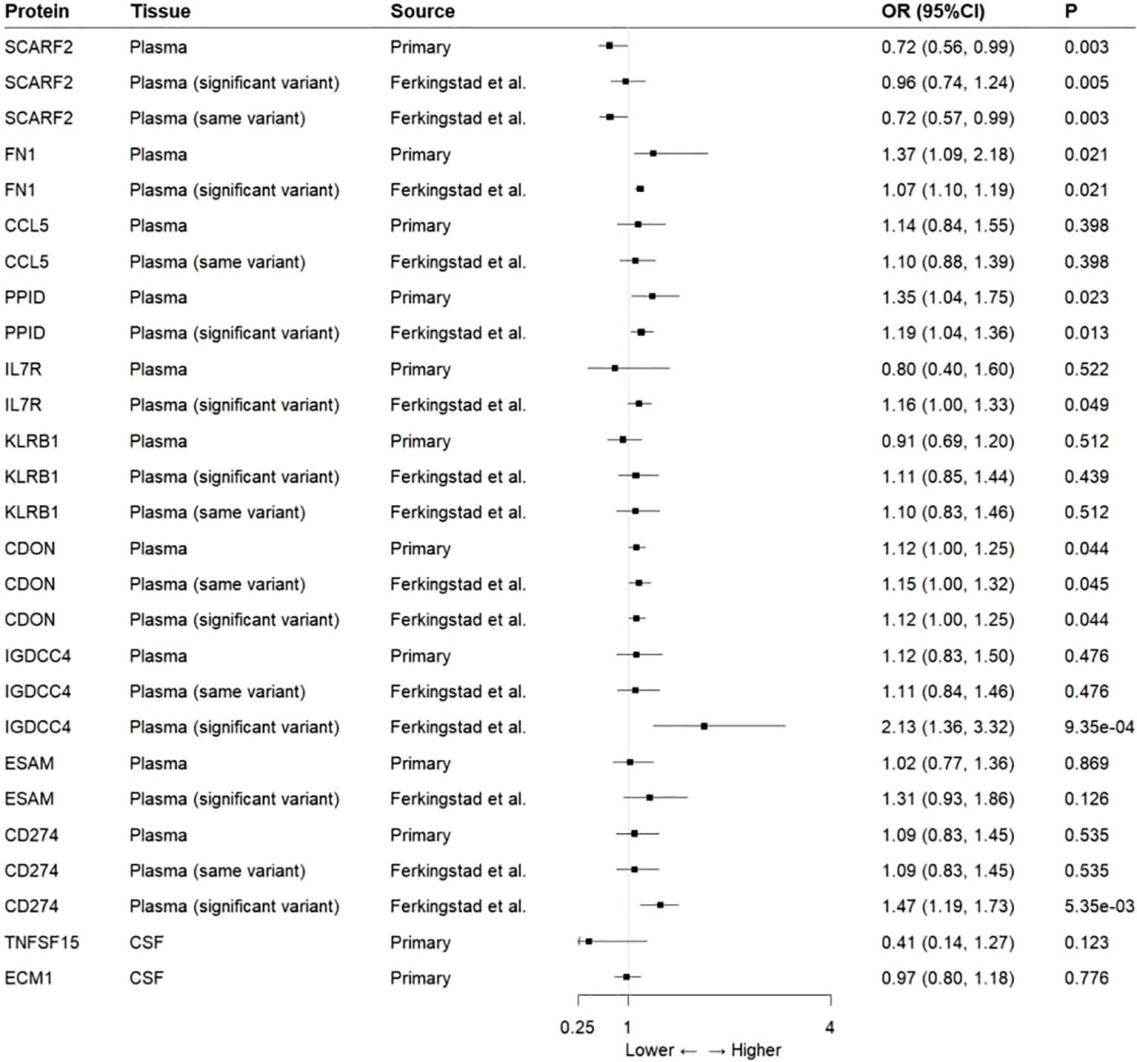
External validation of the causal relationship between twelve potential causal proteins and IPF. The ORs for increased risk of IPF were expressed as per SD increase in plasma protein levels and per 10-fold increase in brain protein levels.

Based on the pQTL data of the primary analysis, the same variation as that in the primary analysis and significant variation in different datasets were searched for external validation, so as to improve the scientificity and rigor of Mendelian randomization study of drug targets. The corresponding plasma pQTL data were extracted from Ferkingstad et al’s study as exposure, and IPF data were extracted from FinnGen database as outcome. The results showed that: SCARF2, FN1, PPID, CDON were also found to be associated with IPF in different databases, IL7R, IGDCC4, CD274 only showed weak causal effects with IPF in FinnGen database, CCL5, KLRB1, ESAM, TNFSF15, ECM1 did not produce any satisfactory results in external validation (**Figure 4**; Additional file 1: **Supplementary Table 6**).

### Validation of key gene expression in pulmonary fibrosis

**Figure 5:**
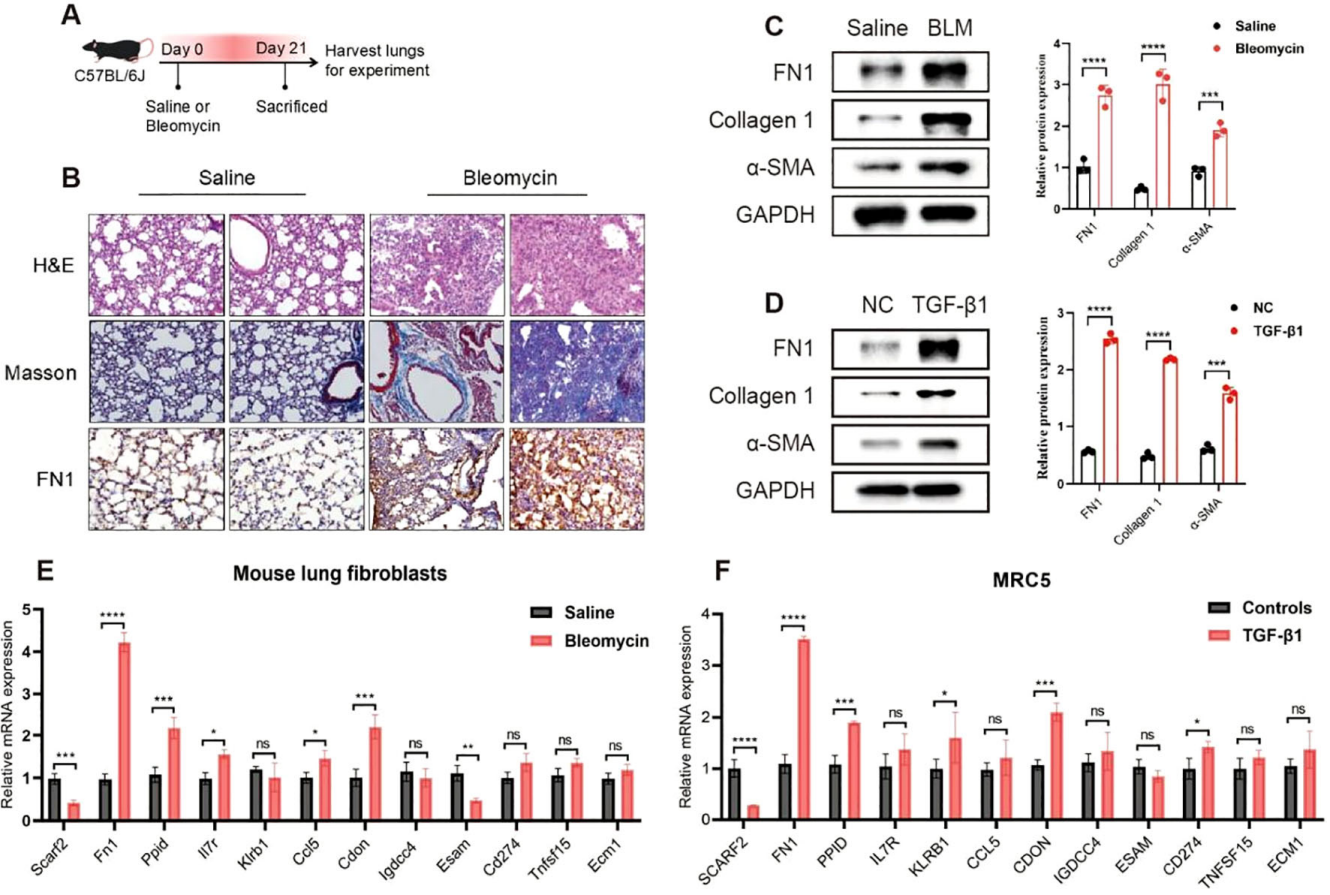
Validation of key gene expression in *in vivo* and *in vitro* experiments. **(A)** Schematic representation of the bleomycin-induced pulmonary fibrosis model. **(B)** Representative lung sections stained with H&E, Masson’s trichrome, and fibronectin immunohistochemistry (IHC) in each group (n = 6; scale bar = 500 μm). **(C)** Western blot (WB) analysis of fibronectin, Col1a1, and α-SMA protein expression in lung tissues from saline- and bleomycin-treated mice. **(D)** WB analysis of fibronectin, Col1a1, and α-SMA protein expression in MRC-5 cells cultured in basal medium or treated with TGF-β1 for 48 hours. **(E)** Quantitative RT-PCR (qRT-PCR) analysis of key gene mRNA expression in lung tissues from saline- and bleomycin-treated mice (n = 6). **(F)** qRT-PCR analysis of key gene mRNA expression in MRC-5 cells cultured in basal medium or treated with TGF-β1 for 48 hours (n = 3).Data are expressed as mean ± standard deviation. *P < 0.05, **P < 0.01, ***P < 0.001, ****P < 0.0001.

To confirm the expression changes of the identified key genes in pulmonary fibrosis, a bleomycin-induced model (2.5 mg/kg) was established. Mice were euthanized on day 21 post-administration, and lung tissues were collected for analysis (**Figure 5A**). Histological examination with H&E and Masson’s trichrome staining showed marked increases in inflammation and collagen deposition in the bleomycin group compared with controls (**Figure 5B**). Western blotting and immunohistochemistry revealed pronounced upregulation of fibrosis markers, including fibronectin, Col1a1, and α-SMA, following bleomycin treatment (**Figure 5B, C**). Quantitative PCR of lung tissue demonstrated significant increases in Fn1, Ppid, Il7r, and Ccl5 mRNA levels, whereas Scarf2 and Esam expression was significantly reduced. No significant differences were detected for Klrb1, Igdcc4, Cd274, Tnfsf15, or Ecm1 between the groups (**Figure 5E**).

*In vitro*, lung fibroblasts were treated with TGF-β1 (10 ng/mL) to induce myofibroblast transformation and extracellular matrix synthesis. Western blot analysis confirmed that TGF-β1 stimulation markedly enhanced fibroblast activation and increased the expression of fibrotic markers (**Figure 5D**). qRT-PCR analysis of 12 key genes showed significant upregulation of FN1, PPID, KLRB1, CDON, and CD274, while SCARF2 expression was significantly decreased. The expression of IL7R, CCL5, IGDCC4, ESAM, TNFSF15, and ECM1 remained unchanged (**Figure 5F**).

Based on the aforementioned evidence, the 12 initially identified proteins were prioritized according to their performance in sensitivity analyses, external validation, and *in vivo* and *in vitro* experiments. Four proteins—SCARF2, FN1, PPID, and CDON—passed all assessments and were designated as primary candidates. The remaining eight proteins did not consistently meet the criteria across sensitivity analyses and external validation and were therefore classified as secondary candidates.

### Comparison of proteins in plasma and cerebrospinal fluid

**Figure 6:**
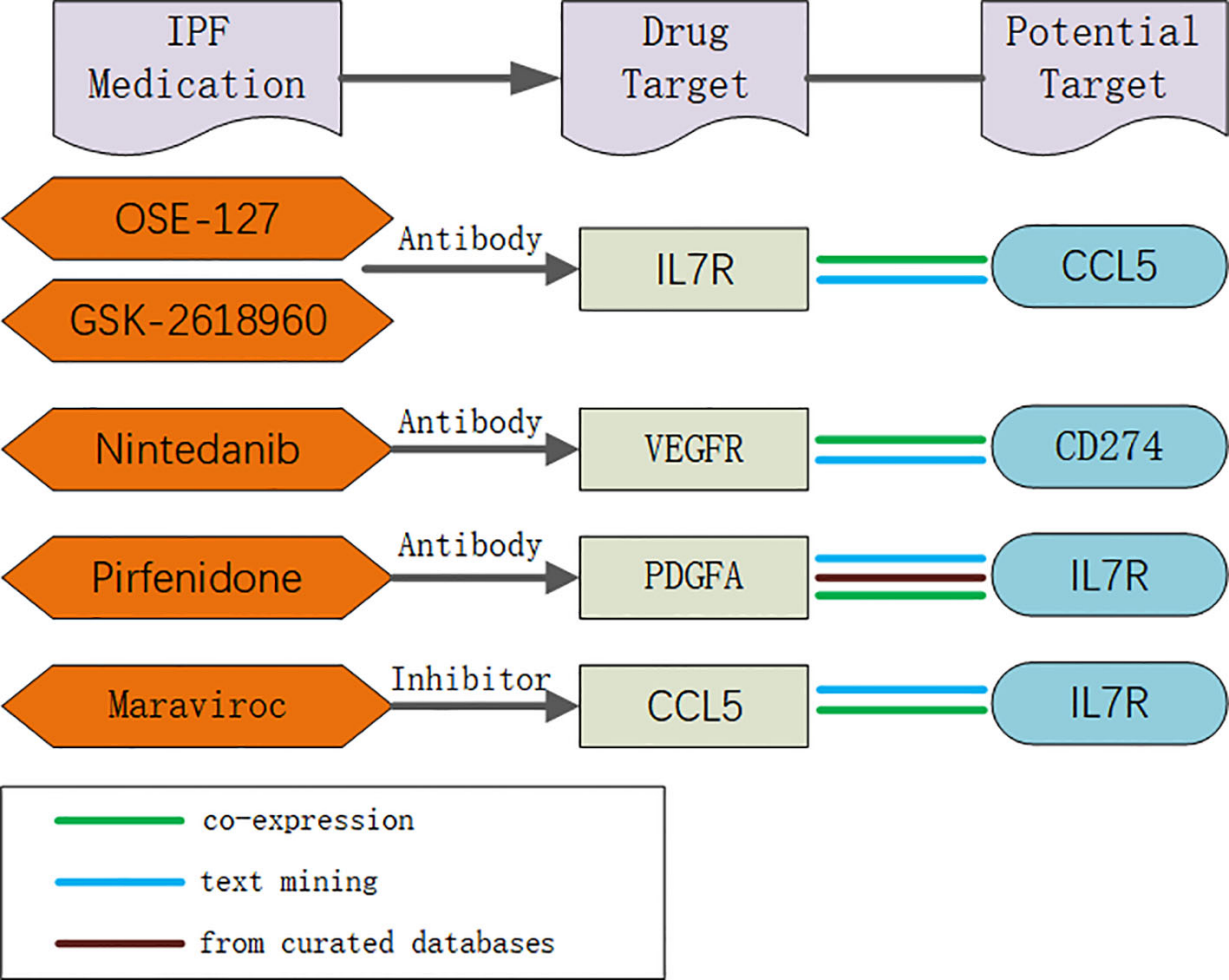
The potential interaction between IPF drug targets and identified potential drug targets.

At the protein level, there was a non-significant positive correlation between CSF and plasma MR results (Spearman correlation coefficient = 0.044). Additionally, when applying various p-value thresholds to restrict the number of proteins included in the analysis, positive correlations persisted but remained statistically insignificant (**Supplementary Figure 3**). The four priority proteins identified through sensitivity analyses and external validation were loaded into the STRING database (https://cn.string-db.org/) to construct protein-protein interaction (PPI) networks, which were subsequently imported into Cytoscape for visualization. The resulting PPI networks illustrate interactions between these four potential drug target proteins and other proteins (**Supplementary Figure 4**). The PPI networks suggested potential links of FN1/CDON with extracellular matrix programs and CCL5/IL7R with immune regulation, but these findings remain exploratory and non-causal.Furthermore, we constructed additional PPI networks incorporating the eight secondary priority proteins alongside the four primary candidates, revealing interactions among these proteins (**Supplementary Figure 5**). Notably, strong and reliable interactions were observed between CDON and FN1, suggesting that CDON may have a similar predictive effect on IPF as FN1. Additionally, CCL5 was found to have synergistic effects with IL7R, which is targeted by Maraviroc, OSE-127, and GSK-2618960 (**Figure 6**), providing robust evidence supporting the potential for dual-target therapies in IPF.

### Association between potential drug targets and current IPF medications

To compare the prioritized proteins with established antifibrotic mechanisms, we constructed PPI network with 12 potential drug target proteins and 2 idiopathic pulmonary fibrosis drug targets (VEGFR, FGFR, PDGFR), loaded the proteins into STRING (https://cn.string-db.org/) database for network creation, and imported the obtained files into Cytoscape for PPI network visualization (**Supplementary Figure 6**). We found significant interactions between ECAM, CCL5, CD274 and VEGFR, and strong interactions between IL7R and FN1 and PDGFA (**Figure 6**). We also searched the Drugbank database for current drugs targeting identified potentially pathogenic proteins (Additional file 1: **Supplementary Table 7**).

## Discussion

To our knowledge, this is the first study to integrate plasma and CSF proteomic data with GEO datasets, using two-sample Mendelian randomization and bioinformatics to investigate pathogenic proteins in IPF. The present framework represents sequential multi-omics prioritization rather than mechanistic systems modeling.From five GEO datasets, 114 upregulated and 142 downregulated DEGs were identified, and their intersection with MR-derived proteins yielded 12 candidates with causal links to IPF risk. Validation through sensitivity analyses, external datasets, and *in vivo* and *in vitro* experiments prioritized these proteins as potential therapeutic targets. These proteins should be interpreted as prioritized candidate proteins rather than experimentally validated therapeutic targets.Among them, SCARF2, FN1, PPID, and CDON were consistently validated as the most promising candidates, while IL7R, IGDCC4, CD274, CCL5, KLRB1, ESAM, TNFSF15, and ECM1 were designated as secondary priorities. In addition, a PPI network was constructed to contextualize these targets and to identify existing drugs associated with them, providing valuable insights for future therapeutic strategies in IPF.

The human genome represents a major therapeutic target; therefore, to identify novel drug targets for IPF, we employed a comprehensive approach combining Mendelian randomization (MR) and bioinformatics analyses to accurately evaluate pathogenic genes and expand potential therapeutic options (37). We implemented a series of rigorous assessments and sensitivity analyses to validate our primary MR findings, ensuring the reliability of the identified drug targets. To minimize bias from horizontal pleiotropy, only cis-pQTLs with significance thresholds below 5×10– (8) were utilized, as these variants directly influence the transcription and translation of related genes (38). Additionally, we excluded weak instrumental variables by calculating the F statistic for each SNP, ensuring all were greater than 10 to confirm strong correlations between instrumental variables and exposure factors (39). A Cochran’s Q test was performed on plasma pQTLs, which revealed no significant heterogeneity or outliers; however, heterogeneity testing could not be applied to CSF proteins due to the presence of only one SNP per protein. Bidirectional MR analyses indicated no evidence of reverse causality for the proteins identified, a conclusion further supported by Steiger filtering (40). Phenotypic scanning revealed associations between these proteins and various traits, including autoimmune diseases, chronic obstructive pulmonary disease, lipid regulation, chronic inflammatory responses, and psychiatric disorders, suggesting potential shared pathogenic pathways. Moreover, Phenoscanner and LDlink analyses showed that KLRB1 and IL7R are associated with chronic inflammation, which is known to play a role in IPF pathogenesis (41). Consequently, while inflammation may influence the relationship between these proteins and IPF, this possibility cannot be fully excluded; thus, interpretations regarding the roles of KLRB1 and IL7R should be made with caution.

Despite advancements in recent years, current therapeutic options for idiopathic pulmonary fibrosis (IPF) remain limited and often unsatisfactory. IPF is incurable, with treatment primarily aimed at symptom management and delaying pulmonary function decline; consequently, greater emphasis should be placed on addressing comorbidities associated with IPF (42, 43). Epidemiological studies indicate that the prevalence of depression among IPF patients ranges from 24.3% to 49.2%, significantly higher than in the general population, thereby exacerbating disease burden and severely impacting daily life (44–46). Furthermore, IPF and depression may share common pathogenic mechanisms, potentially involving neuroinflammation (47) and alterations in neuroplasticity mediated through the blood-brain barrier. Given this, the pathogenic proteins we investigated are relevant not only in plasma but also in cerebrospinal fluid (CSF), warranting a comparative analysis of their subsequent effects. Notably, our study observed discrepancies between proteins identified in plasma and CSF, with no correlation found between the two compartments. This lack of correlation may be attributable to the blood-brain barrier’s selective permeability. Therefore, CSF pQTLs were included as an exploratory extension to capture potential systemic or neuroimmune-related protein signals.Although current evidence remains preliminary, these findings suggest that CSF and plasma represent important pathways for detecting proteins associated with IPF. Proteins identified in CSF, such as TNFSF15 and ECM1, may thus serve as potential therapeutic targets for IPF treatment.

SCARF2 (scavenger receptor class F member 2) is a scavenger receptor protein involved in mediating the binding and degradation of acetylated low-density lipoprotein (LDL) (48). Its tissue expression pattern is similar to that of SCARF1, predominantly occurring in the human heart, lungs, ovaries, and placenta (49). The gene encoding SCARF2 has been associated with various conditions, including van den Ende-Gupta syndrome (VDEGS), glioblastoma, gastric cancer, and mood disorders (50–53). Currently, SCARF2 may reflect scavenger receptor-related immune or lipid-regulatory pathways, but its role in IPF remains unclear. However, Sai Wang et al. (54) utilized multi-omics data to report low plasma SCARF2 protein levels in both COPD and IPF, suggesting a negative correlation between SCARF2 levels and COPD risk. Additionally, another study indicated that higher plasma SCARF2 levels were associated with increased FEV1/FVC ratios (β = 0.300, P = 4.79×10^-21^), implying that elevated SCARF2 might mitigate IPF symptoms (55). The findings from our Mendelian randomization analysis, which demonstrated that increased SCARF2 levels reduce the risk of IPF, alongside these existing data, further support the potential protective role of SCARF2 and validate the reliability of our results.

FN1 is a high-molecular-weight glycoprotein present in the extracellular matrix, serving as a critical component with diverse biological functions. This functional protein is secreted by various cell types, including fibroblasts, vascular endothelial cells, hepatocytes, and vascular smooth muscle cells. It participates in the regulation of cell adhesion, proliferation, differentiation, morphological maintenance, migration, ion exchange, and signal transduction processes (56). Overexpression of FN1 leads to excessive deposition within the cytoplasm, which can impair normal cell functions and promote fibrosis (57). In our study, we observed that elevated plasma FN1 levels may increase the risk of IPF, and external validation using the GEO database further confirmed FN1 as a highly promising target for mitigating IPF risk.FN1 aligns with established extracellular matrix remodeling in IPF, whereas SCARF2 and PPID may highlight less explored immune and senescence-related mechanisms.

Idiopathic pulmonary fibrosis (IPF) is a fatal, age-related lung disease characterized by the pathological accumulation of ECM-secreting myofibroblasts, leading to lung scarring, structural destruction, respiratory failure, and ultimately death (58). Peptidyl-prolyl isomerase D (PPID), also known as Cyclophilin D (CypD), is a recently identified member of the cyclophilin family that resides within the unactivated steroid hormone receptor complex. PPID contains a region homologous to FKBP59, a member of the FK506-binding protein family, which is also part of this receptor complex (59, 60). PPID has been shown to promote transient opening of mitochondrial permeability transition pores (mPTP), and senescent cells depend on cyclophilin D to facilitate calcium overload release from mitochondria, leading to persistent cell aggregation (61). Cell senescence is widely regarded as a critical factor underlying age-related fibroproliferative diseases, including IPF, with PPID playing a role in the development and accumulation of senescent cells. Our study found that plasma PPID levels are positively correlated with IPF risk, suggesting that PPID may serve as a potential therapeutic target for cell senescence-driven fibrosis. Additionally, CDON (cell adhesion molecule-related/down-regulated by oncogenes) belongs to the immunoglobulin (Ig)/fibronectin type III subfamily of cell adhesion molecules (62). Our results indicate that elevated plasma CDON levels are associated with increased IPF risk. However, previous research has demonstrated that CDON deficiency can induce overactivation of the Wnt/β-catenin signaling pathway, leading to cardiac dysfunction and fibrosis (63). This appears inconsistent with our findings, which may reflect differences in the pathogenic mechanisms between pulmonary and myocardial fibrosis. Notably, protein interaction network analyses reveal a strong and reliable interaction between CDON and FN1, indicating that while CDON is a potential target for IPF, its role in fibrosis may be complex and warrants cautious interpretation.CDON may interact with ECM-related programs, potentially including FN1-associated networks, although this remains hypothesis-generating.

Furthermore, IL-7R, IGDCC4, CD274, CCL5, KLRB1, and ESAM were identified in our study as being causally associated with IPF risk. CD274, also known as PD-L1 or B7-H1, is encoded by the PDCDL1 gene located on human chromosome 9 and belongs to the B7 immunoglobulin superfamily (64). Current research indicates that CD274 mediates the immunomodulatory functions of fibroblasts, thereby promoting fibrosis, and directly regulates the activation of fibroblasts and their profibrotic phenotypes (65). Additionally, studies employing tissue spatial transcriptomics combined with single-cell RNA sequencing (scRNA-seq) and gene deconvolution analyses have demonstrated that IL7R, CCL5, and KLRB1 play significant roles in liver fibrosis (66). These proteins offer valuable insights as potential therapeutic targets for pulmonary fibrosis.

Our study has several limitations. First, the protein data incorporated from various studies may vary in measurement methods, potentially introducing bias into the results. Additionally, genetic variants among different IPF patient subtypes may differ, which could impact the generalizability of our findings. Second, in the analysis of protein quantitative trait loci (pQTL), only one cis-acting SNP with genome-wide significance (P < 5×10^-8^) was identified for most proteins. The absence of trans-pQTL data limited the application of pleiotropy tests, heterogeneity assessments, and alternative Mendelian randomization (MR) methods. Notably, most SNPs associated with the primary findings had F statistics greater than 10, indicating adequate statistical power; however, for ECM1, the effect allele frequencies retrieved from matched human genome datasets were close to 0.5, which may affect the reliability of its effect direction, warranting cautious interpretation. Third, our study population was limited to individuals of European ancestry, restricting the applicability of the results to other racial groups. Future research involving diverse populations is necessary to facilitate the translation of these findings into clinical practice. Fourth, further loss- and gain-of-function studies are needed to determine whether SCARF2, PPID, and CDON directly regulate fibroblast activation and extracellular matrix deposition.The current network analysis remains descriptive and does not model pathway activity, regulatory directionality, or intercellular communication. Future studies integrating single-cell, spatial, and perturbational data will be required to construct causal regulatory networks. Fifth, the relatively permissive DEG threshold may reduce biological specificity; therefore, the DEG–MR overlap is now interpreted as an exploratory prioritization step rather than definitive target validation.Finally, A bleomycin-induced pulmonary fibrosis model was established in C57BL/6J mice. As this injury-induced model does not fully reproduce the chronicity, spatial heterogeneity, aging-related biology, or UIP pattern of human IPF, the *in vivo* results should be regarded as supportive rather than definitive IPF-specific validation.

## Conclusion

Our study suggests that there is a causal relationship between the levels of four genetically determined priority proteins (SCARF2, FN1, PPID, CDON) and eight sub-priority proteins (IL7R, IGDCC4, CD274, CCL5, KLRB1, ESAM, TNFSF15, ECM1) and IPF, and that four perfectly validated priority proteins may be attractive drug targets for IPF. However, further research is needed to fully understand the role of these proteins in IPF development and progression.

## Data Availability

The original contributions presented in the study are included in the article/**Supplementary Material**. Further inquiries can be directed to the corresponding authors.
