# Supplementary material for: Identification of potential therapeutic targets for idiopathic pulmonary fibrosis: an integrated multiomics analysis
Source: Front Immunol. 2026 Jun 5;17:1754277. doi: 10.3389/fimmu.2026.1754277 (PMC13279423; doi:10.3389/fimmu.2026.1754277)
Supplement: Supplementary file 2 [file Table2.docx]

Supplementary Figure. S1

 Violin plot of twelve differentially expressed genes identified by screening five GEO datasets（*P<0.05;**P<0.01; ***P<0.001）


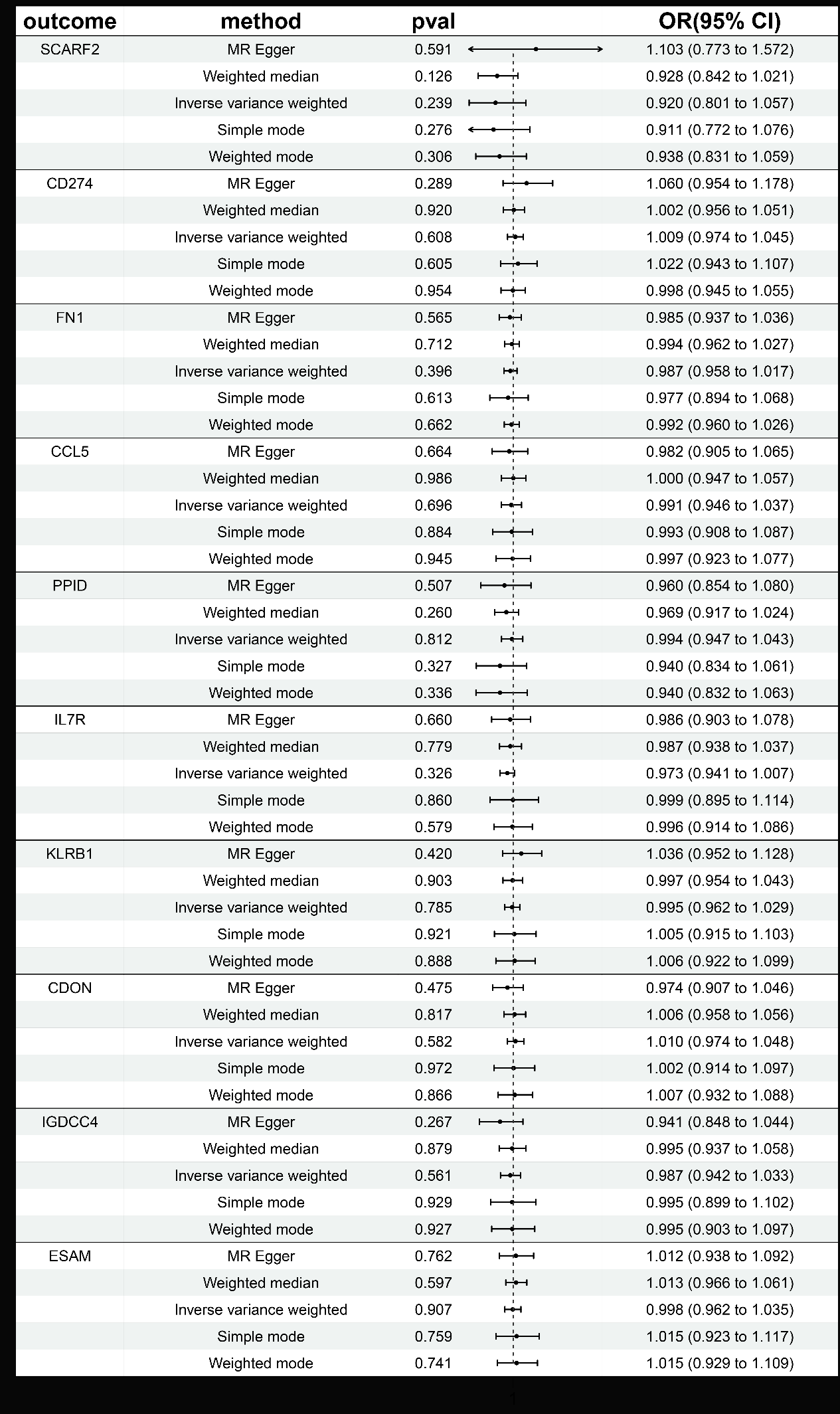


Supplementary Figure. S2 Bidirectional MR analysis for IPF on levels of twelve potential causal proteins

OR stood for the odds ratios for per standard deviation (SD) increase in plasma protein levels.


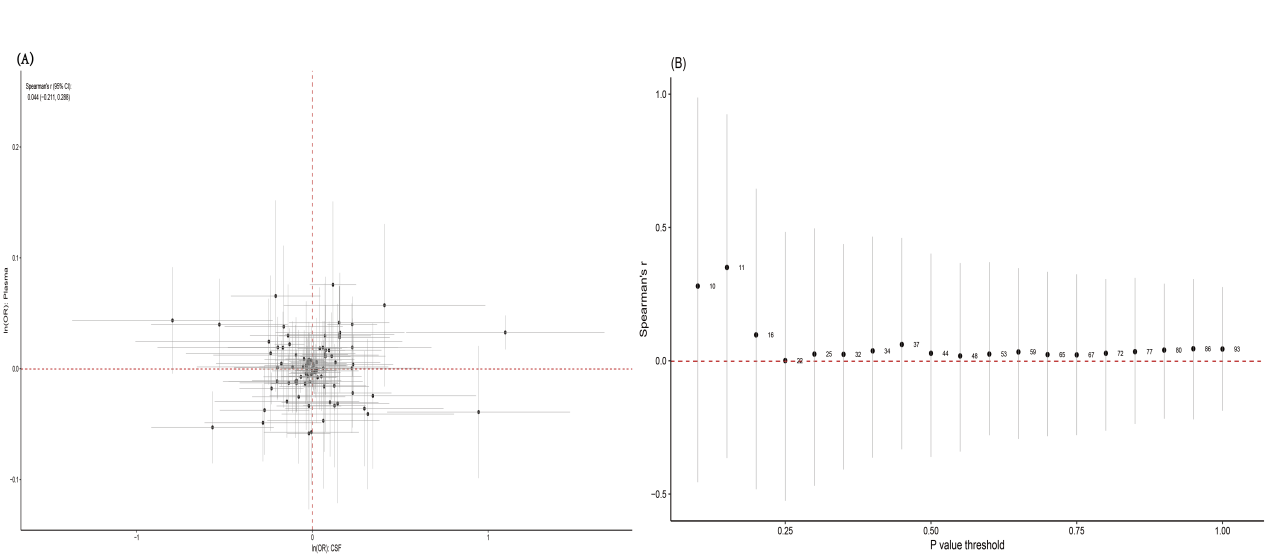


Supplementary Figure. S3 Comparison analysis of MR estimates between plasma proteome and CSF proteome

(A)All 66 overlapping proteins in plasma and CSF were used to perform correlation

analysis. The horizontal and vertical gray line represented the 95% confidence interval

of MR estimates in main analysis. The Spearman correlation coefficient was 0.044 (95% CI: -0.211, 0.288); (B)With different cutoff for P value to include MR estimates,

Spearman correlation coefficient was calculated. The numbers on the left side of the

black point represented the numbers of overlapping proteins correspondingly.


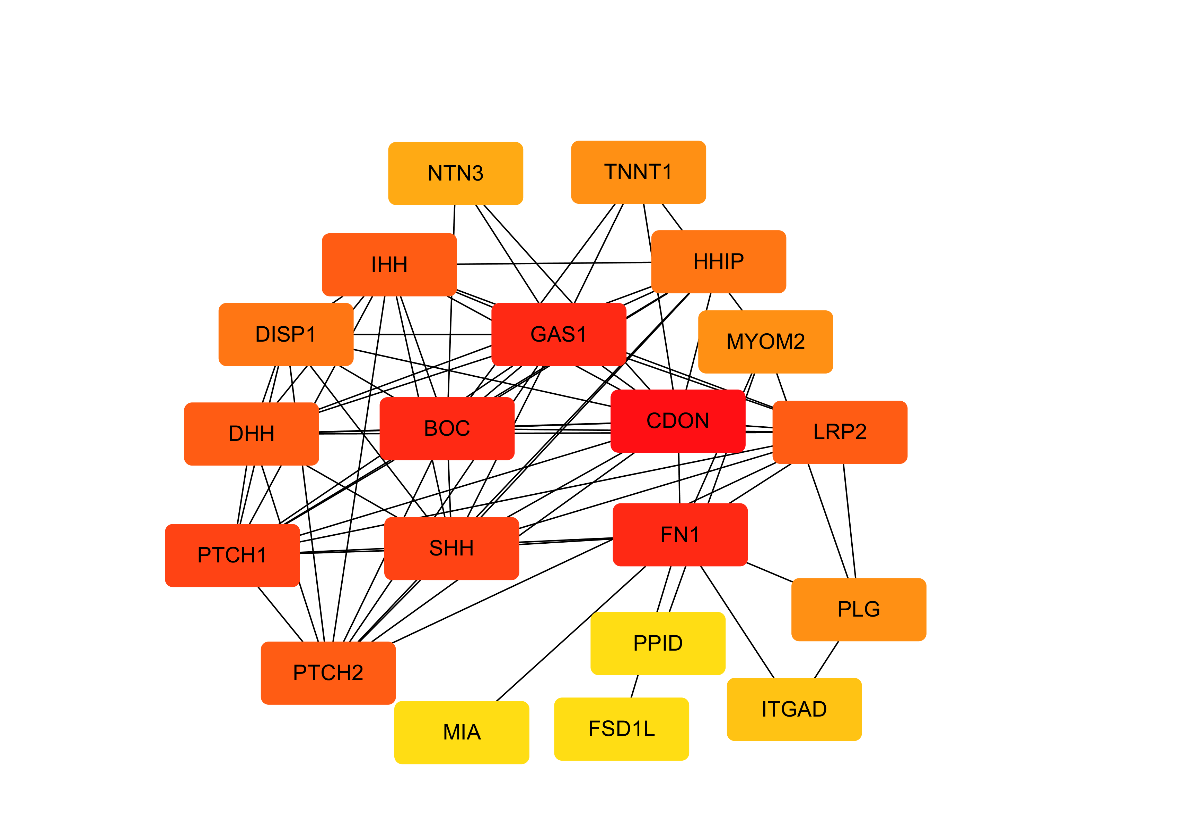
Supplementary Figure. S4 Potential drug target protein-protein interaction network among the suggestive causal proteins (P < 0.05)


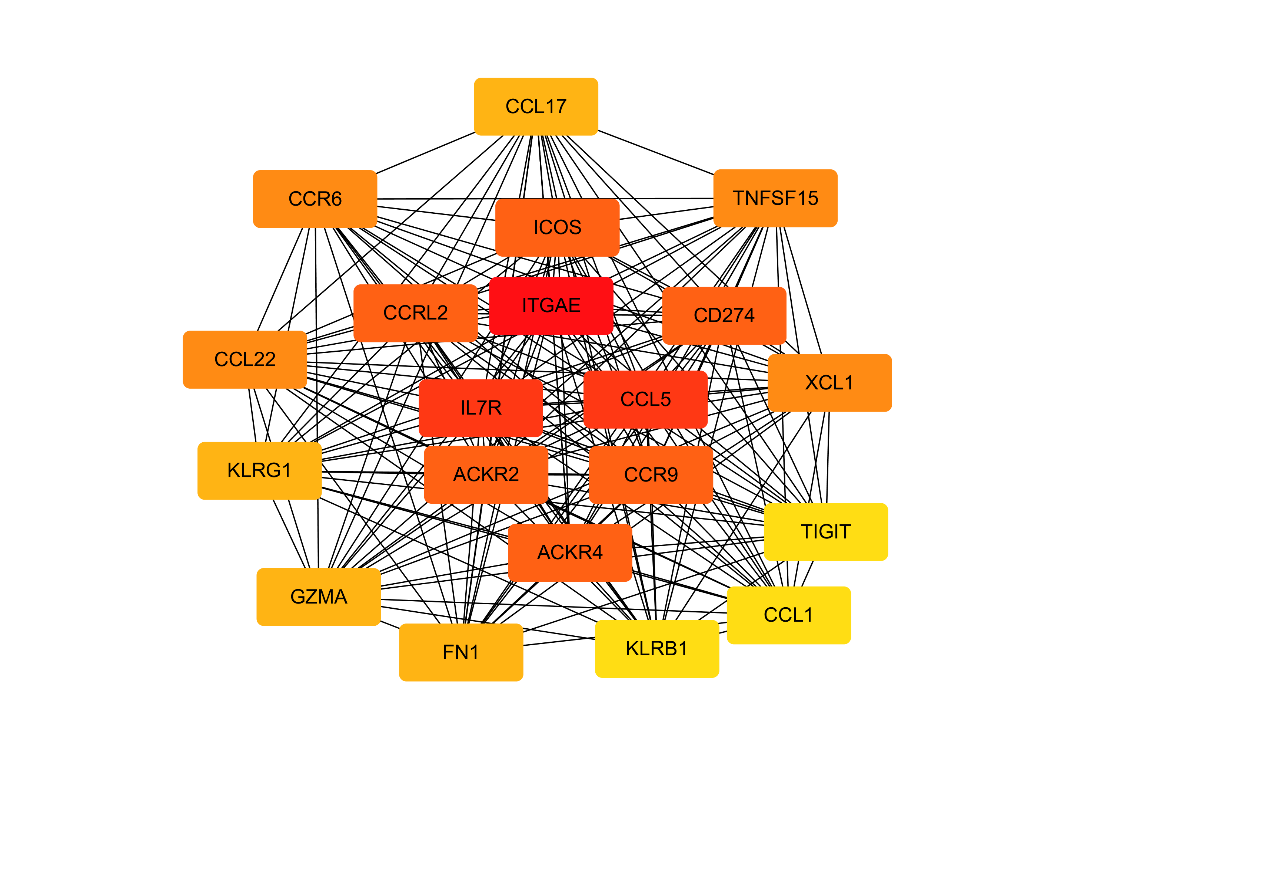
Different red Degrees represent proteins with different degree levels, and the deeper the red degree, the closer the relationship between proteins.

Supplementary Figure. S5 twelve identified protein-protein interaction network among the suggestive causal proteins (P < 0.05)

Different red Degrees represent proteins with different degree levels, and the deeper the red degree, the closer the relationship between proteins.


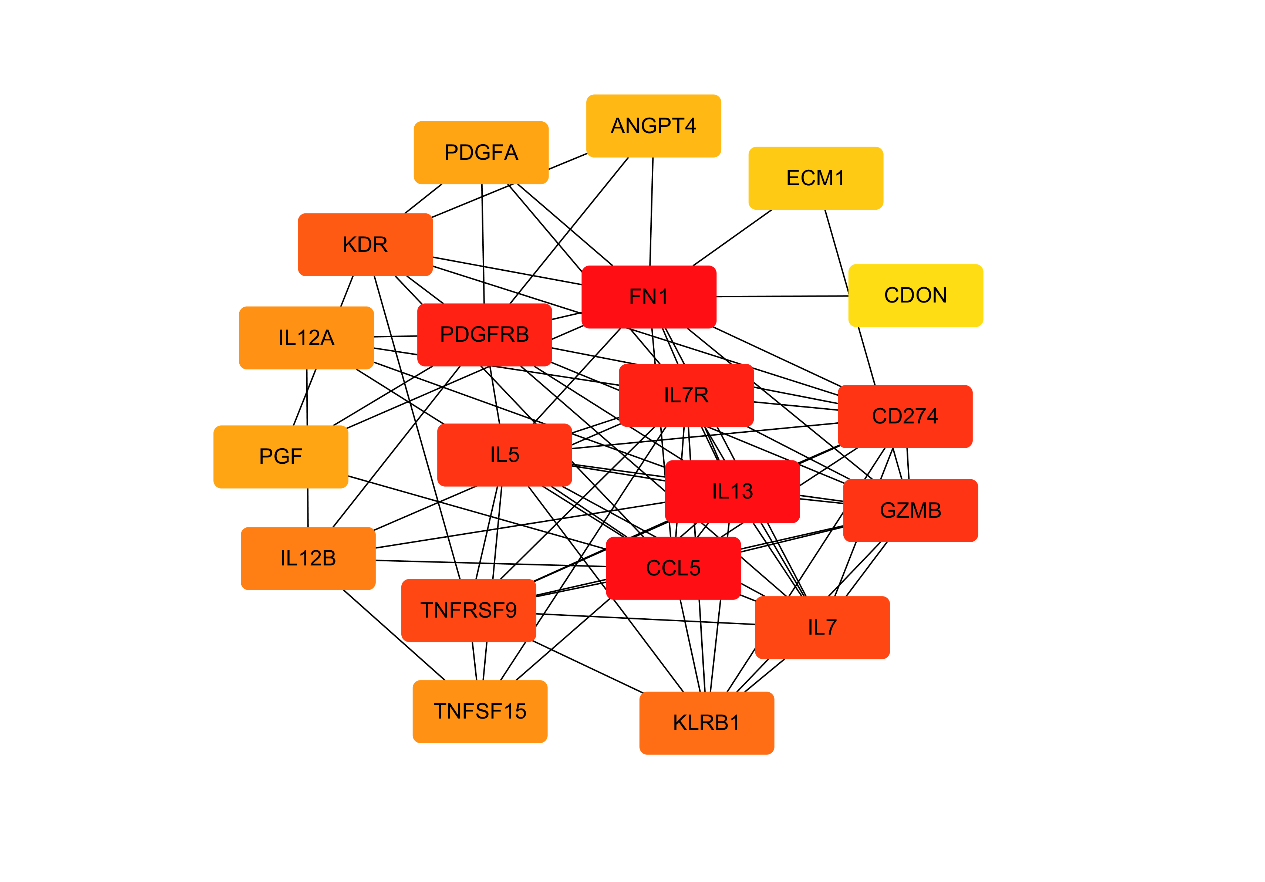


Supplementary Figure. S6 Two IPF drug targets protein-protein interaction network among the suggestive causal proteins (P < 0.05)

Different red Degrees represent proteins with different degree levels, and the deeper the red degree, the closer the relationship between proteins.
